# Calciphylaxis After Kidney Transplant

**DOI:** 10.7759/cureus.4695

**Published:** 2019-05-17

**Authors:** Michael P Ryan, Lindy S Ross

**Affiliations:** 1 Dermatology, University of Texas Medical Branch, Galveston, USA

**Keywords:** caliciphylaxis, kidney transplant, dermatology, wound, renal disease, end stage renal disease, ulceration, ulcer

## Abstract

Calciphylaxis is an uncommon disease that presents with painful ulceration and necrosis of the skin secondary to small vessel calcification and microvascular occlusion. Calciphylaxis carries a poor prognosis as the nonhealing wounds provide a port of entry for pathogens, predisposing these patients to infection and sepsis. Ulcers caused by calciphylaxis are most commonly seen in patients with end-stage renal disease (ESRD) but can also present in patients with normal electrolytes and kidney function. We report a case of a 42-year-old woman with a 10-year history of ESRD who developed rapidly progressing calciphylaxis in her legs and hand, starting three months after successful kidney transplantation. The relationship between kidney transplantation and calciphylaxis remains unclear. There are a handful of cases in which calciphylaxis has been treated by successful kidney transplant, however, other cases have been reported in which calciphylaxis developed after kidney transplantation.

## Introduction

Calciphylaxis, also known as calcific uremic arteriolopathy, is a rare disease consisting of painful, nonhealing cutaneous ulcers, and is most frequently seen in patients with end-stage renal disease (ESRD). In calciphylaxis, calcium deposits within the tunica media of small arterioles, and subsequent thrombosis of these vessels leads to tissue necrosis. The calcification of arterioles can be caused by dysregulation of calcium, phosphate, or parathyroid hormone, which are often abnormal in patients with kidney disease [1]. Calcification alone typically is not enough to create an ulcer; a thrombotic event is often necessary for skin necrosis and ulcer formation. Up to 38% and 43% of patients have been found to be protein C and protein S deficient, respectively [2], and up to 86% of biopsies of calciphylaxis show evidence of thrombosis of dermal arterioles [3].

Despite the predominance of cases in patients with ESRD, calciphylaxis can also be found in patients with normal renal function and normal levels of calcium and phosphate. These cases are often referred to as nonuremic calciphylaxis, a heterogeneous category with several associations. One review found the most common causes of nonuremic calciphylaxis to be hyperparathyroidism (28%), malignancy (22%), alcoholic liver disease (17%), and connective tissue diseases (11%) [2]. The lesions in both nonuremic and uremic calciphylaxis tend to be indistinguishable from each other, initially presenting as tender subcutaneous plaques that progress into nonhealing ulcers with overlying black eschar. Skin changes often begin with a livedo reticularis pattern that can progress to livedo racemosa, and ultimately retiform purpura [1]. Calciphylaxis carries a poor prognosis as the nonhealing ulcers provide an opportunity for secondary infection and sepsis. Mortality rates are high with less than half of patients surviving past one year [1, 3].

The rarity of calciphylaxis as well as the variability in presentation makes calciphylaxis a challenging disease to study. Estimates of prevalence in dialysis patients have ranged as high as 5%, however, true prevalence is difficult to estimate [1]. Even amongst experts in this area, there are significant disagreements on many aspects of diagnosis and management [4]. Many treatments for calciphylaxis have been proposed, and while no blinded randomized studies have taken place for any treatments, sodium thiosulfate either by injection or intravenous administration is one of the most commonly utilized treatments along with stringent wound care regimens [1].

## Case presentation

**Figure 1:**
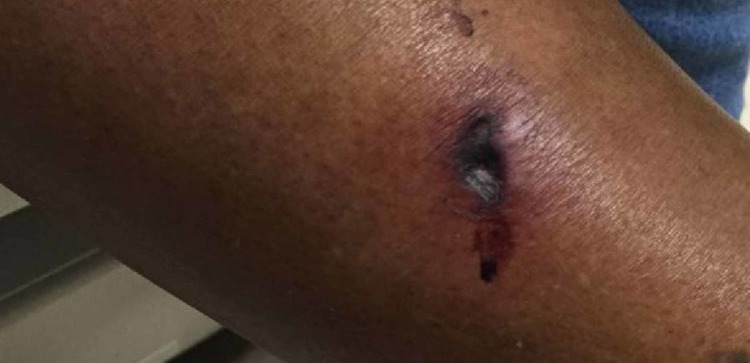
Initial ulcer on patient’s left calf at the time of office visit.

A 42-year-old African American female presented to dermatology clinic for evaluation of a nonhealing painful sore on her left calf (Figure 1). The patient had a history of hypertension, stroke, obesity, seizures, anemia, substance abuse, gout, pheochromocytoma, and ESRD requiring dialysis for the past 10 years. Three months prior, she had received a cadaveric donor kidney and had stable graft function since (creatinine 1-1.5). Before transplantation, her creatinine and phosphorus had risen to 11.5 and 9.0, respectively. Immunosuppressive therapy consisted of prednisone, sirolimus, tacrolimus, and mycophenolate.

Physical examination revealed a violaceous to black retiform plaque that was painful with palpation. The lesion was biopsied with a 4 mm punch in office, however, before the results were available, the patient presented to the emergency room and was admitted to the hospital due to uncontrollable lower extremity pain from the ulcer. Despite initial biopsies that were suggestive of nodular vasculitis or erythema nodosum, there was strong clinical suspicion of calciphylaxis at both the clinic visit and the hospitalization, so treatment with sodium thiosulfate was started, and collagenase ointment was used for wound care. 

**Figure 2:**
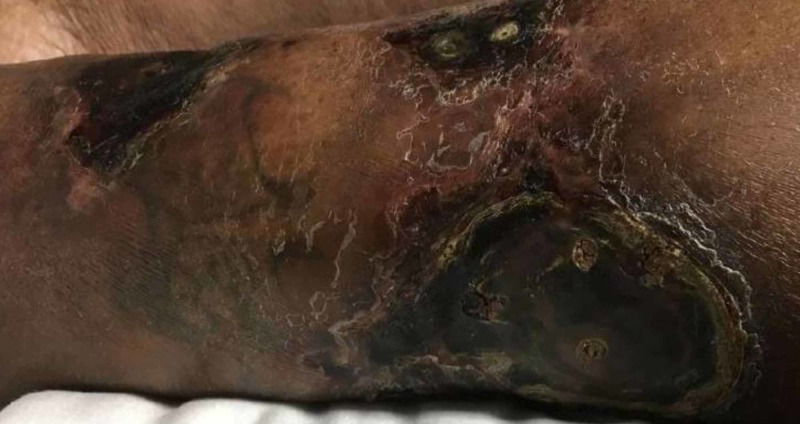
Patient’s left calf three months after initial clinic visit.

**Figure 3:**
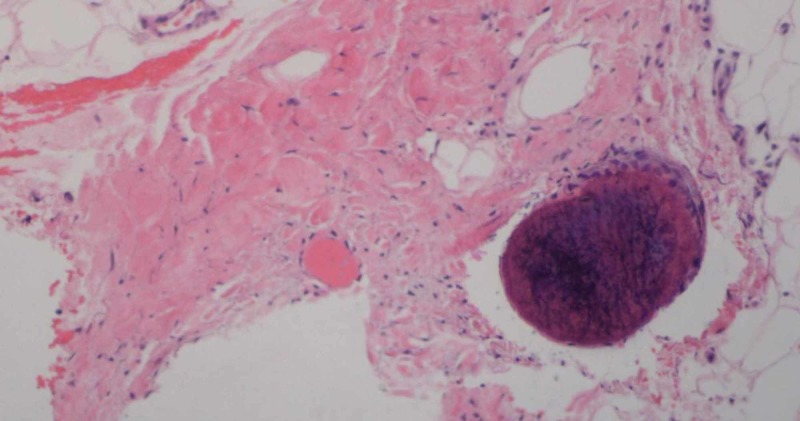
Vascular congestion in the dermis and subcutis with focal calcification (H&E 100x).

Over the next three months, the patient’s ulcer progressed (Figure 2) and additional ulcers appeared on her hand and both feet. She was hospitalized repeatedly for her ulcers and on her first re-hospitalization, another skin biopsy was performed which showed vascular calcification and congestion in the dermal and subcuticular vessels which was consistent with calciphylaxis (Figure 3). The patient sporadically utilized the wound care clinic and received intermittent sodium thiosulfate injections over the course of a year before self-discontinuing. At that time, all other wounds had healed and only the ulcer on her left lower leg remained, which closed six months later.

 

## Discussion

Cutaneous ulcers can have numerous underlying etiologies and many ulcerative diseases can appear clinically similar to calciphylaxis. A careful history is important in differentiating calciphylaxis from ulcers caused by conditions such as warfarin skin necrosis, venous stasis dermatitis, antiphospholipid antibody syndrome, cryoglobulinemia, purpura fulminans, peripheral artery disease, heparin skin necrosis, vasculitis, or trauma [1]. Calciphylaxis should be on the differential for any patient with comorbid ESRD, but diagnosing cases of nonuremic calciphylaxis can be much more challenging and it is important to recognize that it can also occur in patients without electrolyte abnormalities.

Kidney transplantation should theoretically improve or treat ulcers in patients with renal disease as the new kidney acts to restore the mineral balance [1], however, cases such as this one exist in which calciphylaxis arises after kidney transplant. A recent review found 17 cases of calciphylaxis developing after kidney transplant and five cases developing after simultaneous liver-kidney transplant. For the patients who received kidney transplants, 85.7% had ESRD or chronic kidney disease (CKD) at the time of calciphylaxis onset, and the median time to onset was 67 months after transplantation [5]. This case is unique in that the onset of calciphylaxis occurred just six months after transplantation despite the patient having proper graft function.

One potential link between transplantation and calciphylaxis could be the use of systemic corticosteroids in transplant patients. Two articles found that corticosteroid use was present as a predisposing factor in 61% [2] and 80% [3] of patients with nonuremic calciphylaxis. Another explanation for calciphylaxis in transplant patients could be graft failure or loss of function over time, as evidenced by the high proportion of patients with CKD or ESRD at the time of onset. Despite reports of calciphylaxis following kidney transplant, there have been five cases in which patients with calciphylaxis were cured after undergoing renal transplant. These include a case series [6], a case report [7], and an abstract (Abstract: Ullah A, Roy-Chaudhury P, Mogilishetty G, Steve Woodle E, Govil A. Resolution of Calciphylaxis after Successful Kidney Transplantation. NKF Spring Clinical Meetings; 2009).

## Conclusions

This case is unique in that calciphylaxis developed after kidney transplantation despite good graft function. The association between calciphylaxis and ESRD is well known and calciphylaxis should be on the differential for ulcers in patients with ESRD. Calciphylaxis can also present in patients with normal renal function and electrolyte levels and recognizing calciphylaxis in these nonuremic patients can be challenging. The reason for occurrence after transplantation is not completely understood. We recommend that dermatologists keep calciphylaxis in mind when evaluating patients with painful, nonhealing ulcers, even in patients without underlying renal abnormalities.
